# Diabetes Alters KIF1A and KIF5B Motor Proteins in the Hippocampus

**DOI:** 10.1371/journal.pone.0065515

**Published:** 2013-06-12

**Authors:** Filipa I. Baptista, Maria J. Pinto, Filipe Elvas, Ramiro D. Almeida, António F. Ambrósio

**Affiliations:** 1 Centre of Ophthalmology and Vision Sciences, IBILI, Faculty of Medicine, University of Coimbra, Coimbra, Portugal; 2 Pharmacology and Experimental Therapeutics, IBILI, Faculty of Medicine, University of Coimbra, Coimbra, Portugal; 3 Center for Neuroscience and Cell Biology, University of Coimbra, Coimbra, Portugal; 4 PhD Programme in Experimental Biology and Biomedicine (PDBEB), Centre for Neuroscience and Cell Biology, University of Coimbra, Coimbra, Portugal; 5 AIBILI, Coimbra, Portugal; University of Nebraska Medical Center, United States of America

## Abstract

Diabetes mellitus is the most common metabolic disorder in humans. Diabetic encephalopathy is characterized by cognitive and memory impairments, which have been associated with changes in the hippocampus, but the mechanisms underlying those impairments triggered by diabetes, are far from being elucidated. The disruption of axonal transport is associated with several neurodegenerative diseases and might also play a role in diabetes-associated disorders affecting nervous system. We investigated the effect of diabetes (2 and 8 weeks duration) on KIF1A, KIF5B and dynein motor proteins, which are important for axonal transport, in the hippocampus. The mRNA expression of motor proteins was assessed by qRT-PCR, and also their protein levels by immunohistochemistry in hippocampal slices and immunoblotting in total extracts of hippocampus from streptozotocin-induced diabetic and age-matched control animals. Diabetes increased the expression and immunoreactivity of KIF1A and KIF5B in the hippocampus, but no alterations in dynein were detected. Since hyperglycemia is considered a major player in diabetic complications, the effect of a prolonged exposure to high glucose on motor proteins, mitochondria and synaptic proteins in hippocampal neurons was also studied, giving particular attention to changes in axons. Hippocampal cell cultures were exposed to high glucose (50 mM) or mannitol (osmotic control; 25 mM plus 25 mM glucose) for 7 days. In hippocampal cultures incubated with high glucose no changes were detected in the fluorescence intensity or number of accumulations related with mitochondria in the axons of hippocampal neurons. Nevertheless, high glucose increased the number of fluorescent accumulations of KIF1A and synaptotagmin-1 and decreased KIF5B, SNAP-25 and synaptophysin immunoreactivity specifically in axons of hippocampal neurons. These changes suggest that anterograde axonal transport mediated by these kinesins may be impaired in hippocampal neurons, which may lead to changes in synaptic proteins, thus contributing to changes in hippocampal neurotransmission and to cognitive and memory impairments.

## Introduction

Diabetes has been associated with cognitive and memory impairments, indicating that the hippocampus can be affected by this disease. Several studies have demonstrated that diabetes impairs synaptic structure and function in the hippocampus both presynaptically [1], [2] and postsynaptically [3,4]. Previously, we found that diabetes changes the levels of several synaptic proteins involved in exocytosis in hippocampal and retinal nerve terminals, suggesting that axonal transport of those proteins to distal synaptic sites may be impaired under diabetes [2,5]. Moreover, in hippocampal cell cultures, we also found that prolonged exposure to high glucose leads to an accumulation of syntaxin-1, VGluT-1 and synaptotagmin-1 at the cell body of hippocampal neurons, further suggesting that axonal transport may be affected [6]. Potential alterations in axonal transport can somehow contribute to the development of cognitive impairment and memory loss under diabetes.

The impairment of axonal transport is an early and perhaps causative event in many neurodegenerative diseases, and might be due to alterations and/or loss of motor proteins (kinesin and dynein), microtubules, cargoes (by inhibiting their attachment to motor proteins) and ATP fuel supply (mitochondria) which enables molecular motors to undertake the axonal transport [7]. The inhibition of axonal transport leads to a rapid loss of function in the distal axon and to a “dying back” axonal degeneration. The axonal transport is known to be affected in experimental models of diabetes. Most studies regarding nerve dysfunction in diabetes focus on the peripheral nervous system, however increasing evidence also shows that the central nervous system can be affected by diabetes. At peripheral nervous system level, a reduction in retrograde transport has been reported, namely the transport of nerve growth factor in the sciatic nerve of diabetic rats, and endogenous neurotrophins on the cervical and vagus nerve of diabetic rats [8–10]. Moreover, alterations in the axonal caliber in nerves of diabetic animals are likely to be secondary to the impairment of slow anterograde axonal transport, which is correlated with reduced local levels of neurofilament [11,12]. Studies using fluoro-gold labelling showed that diabetes affects the retrograde axonal transport in retinal ganglion cells [13,14], and recently, a deficit in anterograde transport from the retina to the superior colliculus was detected at 6 weeks of diabetes [15]. Furthermore, it was also shown that hyperglycemia impairs axonal transport in olfactory receptor neurons in mice [16]. Nevertheless, to our knowledge, no studies have been performed to analyze the effect of diabetes on axonal transport in the hippocampus, or to investigate local changes in motor proteins in hippocampal neurons. Therefore, the goal of this work, was to evaluate the impact of early diabetes in the hippocampus, namely in the content and distribution of KIF1A (kinesin that transports synaptic vesicle precursors containing synaptophysin and synaptotagmin), KIF5B (kinesin that transports mitochondria and membrane organelles that contain presynaptic membrane proteins such as syntaxin-1 and SNAP-25) and dynein (motor protein responsible for the retrograde axonal transport of organelles, such as mitochondria). Moreover, since hyperglycemia has been considered the main pathogenic factor underlying the development of diabetic complications, we aimed to evaluate whether high glucose *per se*, giving particular attention to changes occurring in the axons, could affect the levels and distribution of motor and synaptic proteins, and the distribution of mitochondria in the axons of hippocampal neurons.

## Experimental Procedure

### Animals

All animals were handled according to the EU guidelines for the use of experimental animals (86/609/EEC), and the experiments were approved by our Institutional Ethics Committee (Comissão de Ética da Faculdade de Medicina da Universidade de Coimbra). Approval ID: FMUC/08/11. Male Wistar rats (Charles River Laboratories), eight weeks-old, were randomly assigned to control or diabetic groups. Diabetes was induced with a single intraperitoneal injection of streptozotocin (STZ; 65 mg/kg, freshly dissolved in 10 mM sodium citrate buffer, pH 4.5) (Sigma, St. Louis, MO, USA). Hyperglycemic status (blood glucose levels exceeding 250 mg/dl) was confirmed two days later with a glucometer (Elite, Bayer, Portugal). Before sacrifice, rats were weighted and blood samples were collected to measure glucose levels. Diabetic rats and age-matched controls were anesthetized with halothane and then sacrificed, two and eight weeks after the onset of diabetes.

### Immunohistochemistry in Brain Slices

#### Brain slices preparation

Rats from each experimental group were deeply anesthetized with ketamine/xylazine and transcardially perfused with 0.1 M phosphate-buffered saline solution (PBS, in mM: 137 NaCl, 2.7 KCl, 4.3 Na_2_HPO_4_, 1.47 KH_2_PO_4_; pH 7.4) followed by 4% paraformaldehyde (PFA) in 0.1 M PBS. The brains were removed and post-fixed for 24 h in 4% PFA and then dehydrated in 20% sucrose in 0.1 M PBS for 24 h. Brain slices (30 µm thickness) were cut in a cryostat (Leica CM3050S, Nussloch, Germany) and collected in 0.1 M PBS with 0.01% sodium azide. Brain slices were used for free-floating immunohistochemistry.

#### Free-floating immunohistochemistry

Slices were washed twice with 0.1 M PBS, blocked with 0.25% Triton X-100 and 5% normal fetal bovine serum (FBS) in 0.1 M PBS for 1 h at room temperature, and then incubated with the appropriate primary antibodies (listed in Table 1) for 24 h at 4°C. Incubation with primary antibodies was followed by incubation with conjugated secondary antibody Alexa Fluor-488 (donkey anti-goat IgG, 1∶250), for sections stained for KIF1A and KIF5B, or Alexa Fluor-568 (goat anti-mouse IgG, 1∶250), for sections stained for dynein, plus DAPI (1∶5,000), to stain cell nuclei, for 2 h 30 min at room temperature. From this point forward, the slices were protected from light. Sections were then washed three times with 0.1 M PBS in the dark and then mounted on slides with glycergel (Dako mounting medium). Sections were examined with a LSM 710 Meta Confocal laser scanning microscope (Zeiss, Germany).

**Table 1:** List of primary antibodies.

Primary Antibody	Sample	Antibody Dilution	Protein (µg)	Source
Mouse anti-KIF1A	Total Extracts Hippocampus	1∶1,000	20	BD Biosciences
	Total Extracts Primary cultures	1∶1,000	80	
Goat anti-KIF1A	Immunocytochemistry	1∶50	_	Santa Cruz
	Immunohistochemistry	1∶50	_	
Goat anti-KIF5B	Total Extracts Hippocampus	1∶2,000	10	Abcam
	Total Extracts Primary cultures	1∶2,000	20	
	Immunocytochemistry	1∶100	_	
	Immunohistochemistry	1∶100	_	
Mouse anti-Dynein	Total Extracts Hippocampus	1∶2,000	20	Abcam
	Total Extracts Primary cultures	1∶2,000	40	
	Immunocytochemistry	1∶100	_	
	Immunohistochemistry	1∶100	_	
Mouse anti-Tau	Total Extracts Primary cultures	1∶1,000	30	Cell Signaling
	Immunocytochemistry	1∶500	_	
Rabbit anti-TUJ-1	Immunocytochemistry	1∶1,000	_	Covance
Mouse anti-SNAP-25	Immunocytochemistry	1∶100	_	SYSY
Mouse anti-Synaptophysin	Immunocytochemistry	1∶50	_	Chemicon
Mouse anti-Syntaxin-1	Immunocytochemistry	1∶100	_	SYSY
Mouse anti-Synaptotagmin-1	Immunocytochemistry	1∶200	_	SYSY

#### Immunofluorescence quantification in hippocampal subregions

A semi-quantitative determination of immunoreactive product densities at the level of the dorsal hippocampus was performed using ImageJ 1.42 software. In order to determine the fluorescence intensity of motor proteins (KIF1A, KIF5B and dynein), slides containg hippocampal slices from control and diabetic groups were blind coded. Sections from each immunohistological experiment, consisting of samples from control and diabetic group, were captured under identical conditions. Typically, four sections from each animal brain were used and the CA1, CA3 and DG subregions were imaged for quantification. Random window sampling within the subregions was carried out for quantification so that the intrinsic variability in the expression was appropriately quantified. To remove tissue background, for each image, a negative control (primary antibody omitted) of coverslipped tissue at the similar location was imaged, and background values were then subtracted from the experimental values, which were expressed in fluorescence arbitrary units (AU). The product densities were averaged across the four sections from each brain and expressed as mean percentage change; the percentage change across the control and diabetic groups was obtained and expressed as mean ± SEM. Although the intensity of staining varied from one experiment to another, within a single experiment the application of primary and secondary antibodies, exposure times and acquisition image settings were uniform. This approach provides a measurement of the relative percentage change among control and diabetic groups based on the density of staining in a given brain region.

### Preparation of Total Hippocampal Extracts

After dissection, the hippocampi from each rat were homogenized in lysis buffer (50 mM Tris-HCl, pH 7.4, 0.5% Triton X-100, supplemented with complete miniprotease inhibitor cocktail tablets and 1 mM DTT). The resulting homogenate was sonicated (4 pulses, 2 seconds each) and then centrifuged at 16,100×*g* for 10 min. All procedure was performed at 4°C. The supernatant was stored at −80°C until use.

### Primary Cultures of Rat Hippocampal Neurons

Primary cultures of rat hippocampal neurons were prepared from the hippocampi of E17–E19 Wistar rat embryos. The hippocampi were dissected under sterile conditions, using a light microscope, in Ca^2+^- and Mg^2+^-free Hank’s solution (in mM: 5.36 KCl, 0.44 KH_2_PO_4_, 137 NaCl, 4.16 NaHCO_3_, 0.34 Na_2_HPO_4_.2H_2_O, 5 glucose, 1 sodium piruvate, 10 HEPES and 0.001% phenol red, pH 7.4). The hippocampi were digested with trypsin (0.06%, 15 min, at 37°C; Gibco Invitrogen, Life Technologies, Scotland, UK), in Ca^2+^- and Mg^2+^-free Hank’s solution. The hippocampi were then washed with Hank’s solution containing 10% fetal bovine serum (Biochrom, Cambridge, UK) to stop digestion. The cells were dissociated in Neurobasal medium (Gibco Invitrogen) supplemented with B27 (1∶50 dilution; Gibco Invitrogen), 0.5 mM glutamine, 25 µM glutamate and 50 µg/ml gentamycin. The cells were plated in six-well plates (8.75×10^4^ cells/cm^2^) or in coverslips (2.25×10^4^ cells/cm^2^) coated with poly-D-lysine (0.1 mg/ml). For experiments in which axon segments were analyzed, neurons were plated in the center of the coverslip (approximately 10,000 cells). Under these conditions, axons grow outward the center (were soma and dendrites are located) and away from the dense neuronal network, where they can be imaged and analyzed independently. The cultures were maintained in a humidified incubator with 5% CO_2_/95% air at 37°C for 14 days. The concentration of glucose in control conditions was 25 mM. After seven days in culture, half of the medium was replaced by fresh medium, and cells were incubated with 25 mM of glucose (yielding a total of 50 mM glucose) or with 25 mM mannitol (plus 25 mM glucose in normal medium), which was used as an osmotic control, and maintained for further seven days.

### RNA Extraction and cDNA Synthesis

Total RNA was isolated using the RNeasy Mini Kit (Qiagen, Germany) according to the manufacturer’s instructions. Briefly, one hippocampi, from control and diabetic rats, was mechanically disrupted using a lysis buffer and subsequently homogenized in a QIAshredder homogenizer. The sample was then transferred to an RNeasy spin column, to yield a RNA-enriched solution. RNA concentration was measured using a NanoDrop ND-1000 Spectrophotometer (NanoDrop Technologies, USA) in 2 µL volume. First strand cDNA synthesis was performed using random primers, 0.5 µg total RNA and SuperScript II Reverse Transcriptase (Life Technologies, USA), according to the manufacturer’s instructions. Additionally, the resulting cDNA (2 µl) was subjected to a 35-cycle polymerase chain reaction (PCR) amplification using 2× MyTaq Red Mix (BIOLINE, UK), 200 nM of forward (GCTCCTCCTGAGCGCAAG) and reverse (CATCTGCTGGAAGGTGGACA) β-actin primers. PCR products were visualized after electrophoresis on 1.5% (w/v) agarose gels containing 0.005% (v/v) EtBr in 1× TAE buffer to evaluate genomic DNA contamination (data not shown).

### Primer Design and Evaluation

Primers for quantitative real time polymerase chain reaction (qRT-PCR) were designed using the Beacon Designer 6 software (PREMIER Biosoft International, USA) for the amplification of gene fragments between 70–110 bp in length and an annealing temperature of 56°C. An intron-spanning amplicon was chosen in order to avoid amplification of genomic DNA in the cDNA samples. Amplification efficiency of target and reference genes was evaluated using a cDNA ten-fold dilution series and plotting threshold cycle (Ct) values against cDNA dilution (data not shown). Furthermore, a melting curve was performed at the end of the cycling program to assess the primer specificity, represented by a single peak at the melting temperature of the PCR-product. Primers with amplification efficiency outside of 90–110% range or primer pairs generating multiple peaks were discarded. Final primer sequences and amplicon lengths are shown in Table 2.

**Table 2:** Primer sequences.

Gene	Forward primer (5′-3′)	Reverse primer (5′-3′)	Amplicon size (bp)
**Reference genes**			
GAPDH	GACTTCAACAGCAACTCC	GCCATATTCATTGTCATACCA	105
HPRT	ATGGGAGGCCATCACATTGT	ATGTAATCCAGCAGGTCAGCAA	77
YWHAZ	CAAGCATACCAAGAAGCATTTGA	GGGCCAGACCCAGTCTGA	76
**Target genes**			
KIF1A	CATTAGTTAGTGGCGTTGA	TACCTGGAGGCATTAGAAA	91
KIF5B	GTGATGATTGCGTCCAAG	CTTCTTTGCACAATCGTTG	90
DYNEIN	TTCTGGCGTAGTCCTATT	ACACCACATCTCAAGTCT	104

GAPDH - glyceraldehyde-3- phosphate dehydrogenase;

HPRT - human hypoxanthine phosphoribosyltransferase;

YWHAZ - tyrosine 3-monooxygenase/tryptophan 5-monooxygenase activation protein, zeta polypeptide;

KIF1A - kinesin family member 1A;

KIF5B - kinesin family member 5B;

DYNEIN - dynein cytoplasmic 1 intermediate chain.

### Quantitative Real Time Polymerase Chain Reaction

qRT-PCR was performed using 20 µL total reaction volume containing 10 µL 2× iTaqTM SYBR® Green Supermix with ROX (BioRad, USA), 200 nM of forward and reverse primers and 2 µL of 1∶2 diluted cDNA in a StepOne Plus system (Life Technologies, USA). PCR conditions were: 10 min 95°C for initial denaturation; 40 x 15 sec 95°C for denaturation, 45 sec 56°C for primer annealing and 30 sec 72°C for elongation. Furthermore, at the end of the PCR a melting curve analysis was performed to evaluate unspecific products and primer-dimer formation. Three technical replicates for each biological replicate per group were performed. A non-template control was included for each gene. Ct values were obtained during the exponential amplification phase using automatic threshold option in StepOne Software (Life Technologies, USA).

### qRT-PCR Data Analysis

Reference gene expression stability between different groups was evaluated using the NormFinder analysis algorithm for Microsoft Excel [17], which identified Ywhaz as the most stable gene (stability value: 0.002). Ywhaz gene was selected as our reference gene for normalization of axonal transport protein gene expression in all groups. Relative gene expression data was analyzed using 2^−ΔΔCt^ method [18], where ΔΔCt = (Ct gene of interest-Ct reference gene) diabetes group - (Ct gene of interest-Ct reference gene) control group. The data analysis was based on 5 independent biological replicates per group. The results were expressed as the mean ± SEM. Data were analyzed by the unpaired Student's *t*-test (IBM SPSS Statistics, USA) to determine differences in gene expression between groups. Differences were considered statistically significant when the *p*<0.05.

### Preparation of Extracts of Cultured Hippocampal Neurons

Cells were rinsed twice with ice-cold PBS and then lysed with RIPA buffer (50 mM Tris-HCl, pH 7.4, 150 mM NaCl, 5 mM EDTA, 1% Triton X-100, 0.5% DOC, 0.1% SDS, 1 mM DTT) supplemented with complete miniprotease inhibitor cocktail tablets and phosphatase inhibitors (10 mM NaF and 1 mM Na_3_VO_4_). The lysates were incubated on ice for 30 min and then centrifuged at 16,100×g for 10 min at 4°C. The supernatant was collected and stored at −80°C until use.

### Western Blot Analysis

The protein concentration of each sample was determined by the bicinchoninic acid (BCA) protein assay (Pierce Biotechnology, Rockford, IL, USA). The samples were denaturated by adding 6× concentrated sample buffer (0.5 M Tris, 30% glycerol, 10% SDS, 0.6 M DTT, 0.012% bromophenol blue) and heating for 5 min at 95°C. Equal amounts of protein were loaded into the gel and proteins were separated by sodium dodecyl sulphate-polyacrylamide gel electrophoresis (SDS-PAGE), using 6–8% gels. Then, proteins were transferred electrophoretically to PVDF membranes (Millipore, Billerica, Massachusetts, USA). The membranes were blocked with 5% low-fat milk in Tris-buffered saline (137 mM NaCl, 20 mM Tris-HCl, pH 7.6) containing 0.1% Tween-20 (TBS-T) for 1 h at room temperature. The membranes were then incubated with primary antibodies (listed in Table 1) overnight at 4°C. After washing for 1 h in TBS-T with 0.5% low-fat milk, the membranes were incubated with an anti-mouse or anti-goat alkaline phosphatase-linked IgG secondary antibody (1∶10,000; GE Healthcare, Buckinghamshire, UK) in TBS-T with 1% low-fat milk for 1 h at room temperature. The membranes were processed for protein detection using the enhanced chemifluorescence substrate (ECF; GE Healthcare). Fluorescence was detected on an imaging system (Thyphoon FLA 9000, GE Healthcare) and the digital quantification of bands immunoreactivity was performed using ImageQuant 5.0 software (Molecular Dynamics, Inc., Sunnyvale. CA, USA). The membranes were then reprobed and tested for β-actin immunoreactivity (1∶5,000) or β-III tubulin (1∶5,000) to prove that similar amounts of protein were applied to the gels.

### Immunocytochemistry

Hippocampal cell cultures were washed three times with PBS and fixed with 4% paraformaldehyde and 4% sucrose for 10 min at room temperature. Cells were then washed three times with PBS and permeabilized with 0.25% Triton X-100 in PBS for 5 min at room temperature. Non-specific binding was prevented incubating cells with 3% BSA/0.2% Tween-20 in PBS for 30 min. Cells were then incubated with the primary antibodies (listed in Table 1) for 2 h at room temperature. After incubation, cells were rinsed three times with PBS and incubated with the secondary antibodies for 1 h at room temperature in the dark. The nuclei were stained with DAPI (1∶5,000). Upon rinsing three times with PBS, the coverslips were mounted on glass slides using Dako Fluorescent mounting medium (Dako, Denmark). The preparations were visualized under a confocal laser scanning microscope LSM 710 META (Zeiss, Germany) or under an inverted microscope Zeiss Axiovert 200 (Zeiss, Germany), as indicated in the corresponding figure legends. Neurons were plated at the center of the coverslip to concentrate cell bodies in a limited region, allowing the axons to grow out of this area. This made possible to acquire two sets of images, an area comprising the cell bodies, dendrites and axons and another area with isolated axons (images of this were taken where only tau-positive neurites were present). Quantitative analysis of immunocytochemistry data was performed using ImageJ 1.42 software. In order to determine the ratio between the fluorescence intensity of motor proteins (KIF1A, KIF5B and dynein) and the area of tau fluorescence, 8-bit images (10 per condition) were randomly selected and each channel manipulated separately. Background signal of each channel image was subtracted by adjusting the minimum grey value of the greyscale (greyscale adjustment was equal for all images in each single experiment). Channel images were then thresholded and the signal of the motor proteins expressed in intensity per area (in µm^2^) of tau fluorescence (threshold values were conserved in single experiments). For each image, the ratio between the fluorescence intensity of motor proteins (KIF1A, KIF5B or dynein) and the area of tau fluorescence was calculated.

When looking into the distribution and content of motor and synaptic proteins, specifically in axons, quantitative analysis was performed for images taken to isolated axons only. Quantification of the ratio between motor proteins fluorescence intensity (KIF1A or KIF5B) and the area of tau was performed as described above. In order to determine the number of accumulations, both for KIF1A and synaptic proteins, per area of tau or tuj-1, respectively, 8-bit images (12 per condition) were randomly selected. For each protein of interest, background signal was subtracted (as described before) and threshold values were applied so that only accumulations are taken in consideration (threshold values were conserved in single experiments). Particle analysis was performed and the number of accumulations per axonal area calculated for each image.

The values obtained for each image were normalized against the control mean of that single experiment and for all analysis, results are presented as normalized values ± SEM of the number of images analyzed (10–12 per condition in each experiment). The number of independent experiments is indicated above the graphs.

### Statistical Analysis

For Western blotting analysis, statistical comparisons between diabetic animals and respective age-matched controls were performed using the unpaired Student’s *t*-test (variance analysis was not undertaken since the effect of age on the content of motor proteins was not the aim of this study). Thus, gels were always loaded with samples from age-matched animals and not from animals with different ages.

In order to quantitatively analyze immunofluorescence in brain slices, all values were compiled for statistical analysis and significant difference between control and diabetic animals was performed using the unpaired Student’s *t*-test. Statistical analysis for data obtained from hippocampal cell cultures was performed in Graph Pad Prism 5 software. Statistical significance was assessed by one-way ANOVA analysis followed by Dunnett’s post hoc test or unpaired Student's *t*-test. Differences were considered significant for *p*<0.05.

## Results

### Animals

Before diabetes induction, the body weight of animals assigned for control and diabetic groups was similar (257.7±3.9 g for control animals and 255.3±3.6 g for diabetic animals). The glucose levels were also similar in both groups (87.1±1.2 mg/dl for controls and 86.3±3.7 mg/dl for diabetic animals). Average weight and blood glucose levels for both diabetic and aged-matched control rats at the time of death are given in Table 3. A marked impairment in weight gain occurred in diabetic rats comparing to age-matched controls in all time points analyzed. Diabetic animals also presented significantly higher blood glucose levels when compared to age-matched controls.

**Table 3:** Average weight and blood glucose levels of diabetic and aged-matched control rats.

Diabetes duration	Weight (g)	Blood Glucose (mg/dL)
**2 Weeks**		
Control	327.6±4.9	104.4±3.2
Diabetic	224.4±8.4***	379.4±18.7***
**8 Weeks**		
Control	399.7±10.3	90.2±2.0
Diabetic	247.7±10.3***	498.1±28.6***

Measurements were made immediately before the sacrifice of the animals. ***p<0.001.

### Diabetes Increases mRNA Expression, Protein Levels and Immunoreactivity of KIF1A and KIF5B in the Hippocampus at 8 Weeks of Diabetes

**Figure 1:**
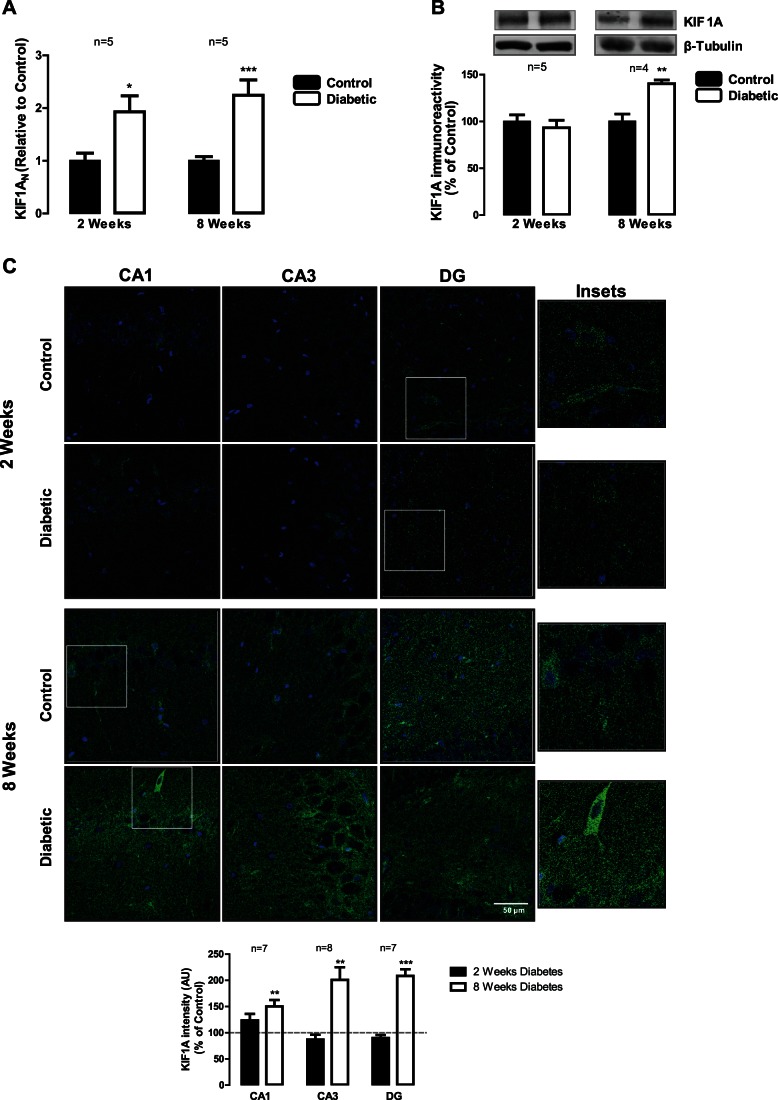
Diabetes induces changes in kinesin KIF1A in the hippocampus. (A) Diabetes upregulates KIF1A mRNA expression, as assessed by RT-qPCR. The results are expressed relatively to age-matched controls, and data are presented as mean ± SEM of 5 animals. *p<0.5; ***p<0.001 compared to age-matched control analyzed by the unpaired Students *t*-test. (B) KIF1A protein levels were analyzed by immunoblotting in total extracts of hippocampus isolated from control and STZ-induced diabetic animals (2 and 8 weeks of diabetes). Representative Western blots are presented above the graphs, with the respective loading controls (β-III tubulin), to confirm that identical amounts of protein from control and diabetic samples were loaded into the gel. The results are expressed as percentage of age-matched controls, and data are presented as mean ± SEM of 4–5 animals. **p<0.01 compared to age-matched control using Student’s *t*-test. (C) The distribution of KIF1A protein in hippocampal subregions of control and STZ-induced diabetic animals was also analyzed by immunohistochemistry. KIF1A immunoreactivity increased at 8 weeks of diabetes. The preparations were visualized under a laser scanning confocal microscope LSM 710 META (Zeiss, Germany). Scale bar: 50 µm. Insets shows the expression pattern of KIF1A in hippocampal slices. The quantification of immunoreactivity (fluorescence intensity arbitrary units) was performed and presented below the images. **p<0.01, ***p<0.01, significantly different from control as determined by the unpaired Student’s *t*-test.

KIF1A is an anterograde motor protein that transports membranous organelles along axonal microtubules [19]. It is thought that this protein may play a critical role in the development of axonal neuropathies, which may result from impaired axonal transport. Our previous results suggest that hyperglycemic conditions might impair axonal transport in hippocampal neurons [2,6]. To our knowledge, the effect of diabetes on the expression and content of motor proteins in the hippocampus has never been addressed, and so we analyzed mRNA levels and the content of KIF1A and KIF5B in hippocampal total extracts by western blotting, as well as the immunoreactivity of both proteins in hippocampal *Cornu Ammonis* (CA1 and CA3) and dentate gyrus (DG) subregions. Increased mRNA expression of KIF1A (increase to 1.92±0.6 of the control) and KIF5B (increase to 1.39±0.2 of the control) were detected after 2 weeks of diabetes. Nevertheless, no significant changes were detected neither in the levels of KIF1A in hippocampal total extracts, nor in its immunoreactivity in hippocampal subregions at 2 weeks of diabetes (Figure 1A and B). At 8 weeks of diabetes, KIF1A mRNA levels significantly increased to 2.24±0.6 of the control, as well as KIF1A protein levels in hippocampal total extracts (increase to 140.3±4.0%) compared to age-matched control animals (Figure 1A and B, respectively). A significant increase in the immunoreactivity of this protein was also observed by immunohistochemistry at 8 weeks of diabetes in CA1, CA3 and DG hippocampal subregions (increase to 150.3±12.3%, 200.9±24.2% and 208.4±12.8% of the control, respectively; Figure 1C).

**Figure 2:**
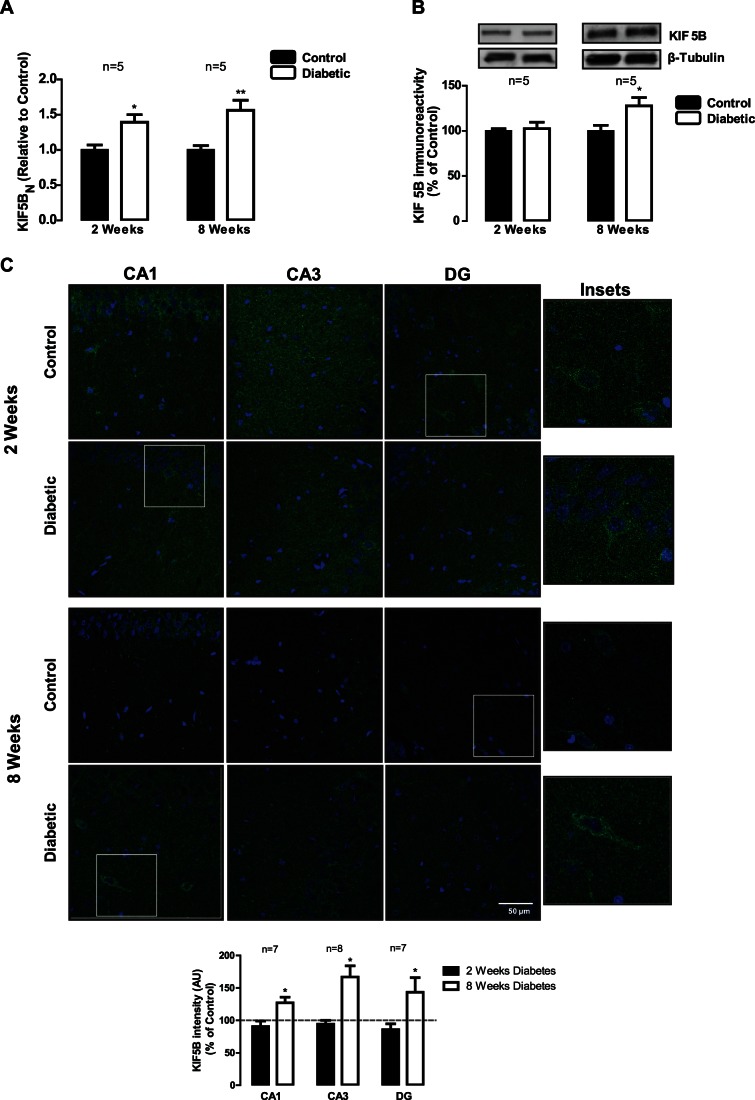
Diabetes induces changes in KIF5B in the hippocampus. (A) Diabetes upregulates KIF5B mRNA expression, as assessed by RT-qPCR. The results are expressed relatively to age-matched controls, and data are presented as mean ± SEM of 5 animals. *p<0.5; **p<0.01 compared to age-matched control and analyzed by the unpaired Students *t*-test. (B) KIF5B protein levels were analyzed by immunoblotting in total extracts of hippocampus isolated from control and STZ-induced diabetic animals (2 and 8 weeks of diabetes). Representative Western blots are presented above the graphs, with the respective loading controls (β-III tubulin), to confirm that identical amounts of protein from control and diabetic samples were loaded into the gel. The results are expressed as percentage of age-matched controls, and data are presented as mean ± SEM of 5 animals. *p<0.05 compared to age-matched control using Student’s *t*-test. (C) The distribution of KIF5B protein in hippocampal subregions of control and STZ-induced diabetic animals was also analyzed by immunohistochemistry. KIF5B immunoreactivity increased at 8 weeks of diabetes. The preparations were visualized under a laser scanning confocal microscope LSM 710 META (Zeiss, Germany). Scale bar: 50 µm. Insets shows the expression pattern of KIF5B in hippocampal slices. The quantification of immunoreactivity (fluorescence intensity arbitrary units) was performed and presented below the images.*p<0.05, significantly different from control as determined by the unpaired Student’s *t*-test.

KIF5B is a microtubule-dependent motor protein required for normal distribution of presynaptic cargoes and mitochondria. At 2 weeks of diabetes, KIF5B mRNA levels were also increased (increase to 1.39±0.3), but no significant changes were observed in KIF5B immunoreactivity in the hippocampus by Western blotting (Figure 2A and B). However, at 8 weeks of diabetes, the mRNA expression significantly increased to 1.56±0.3, and KIF5B protein levels increased to 127.7±9.3% of the control (Figure 2A and B). Moreover, by immunohistochemistry, it was also detected an increase in the immunoreactivity of this protein in CA1, CA3 and DG hippocampal subregions (increase to 127.1±8.5%, 166.9±17.3% and 143.4±22.4% of the control, respectively; Figure 2C).

### Diabetes does not Affect the Content of Dynein in the Hippocampus

**Figure 3:**
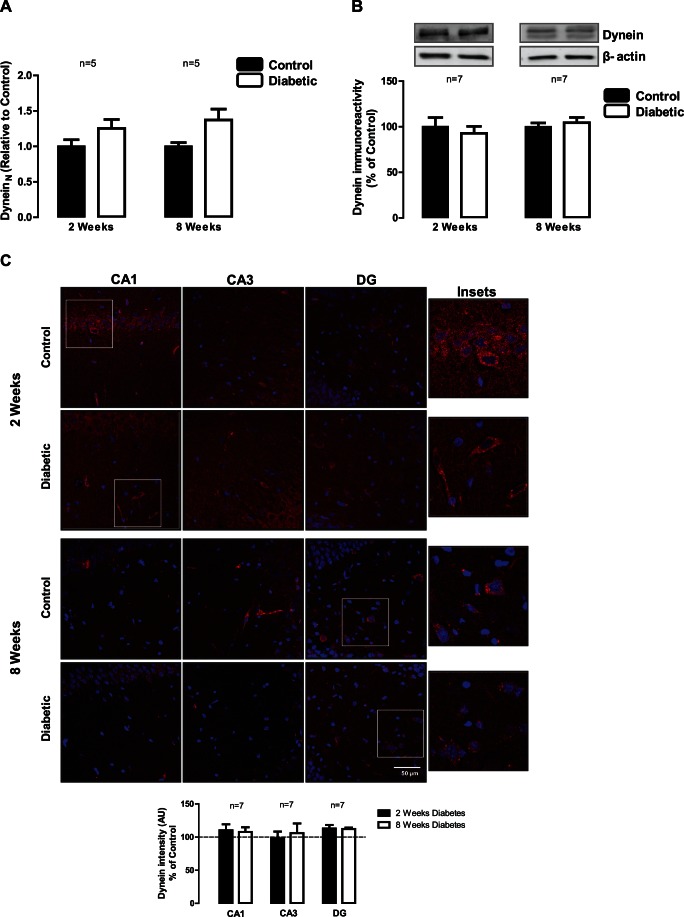
Diabetes does not induce changes in dynein in the hippocampus. (A) Diabetes does not change dynein mRNA expression, as assessed by RT-qPCR. The results are expressed relatively to age-matched controls, and data are presented as mean ± SEM of 5 animals as assessed by the unpaired Students *t*-test. (B) Dynein protein levels were analyzed by immunoblotting in total extracts of hippocampus isolated from control and STZ-induced diabetic animals (2 and 8 weeks of diabetes). Representative Western blots are presented above the graphs, with the respective loading controls (β-III tubulin), to confirm that identical amounts of protein from control and diabetic samples were loaded into the gel. The results are expressed as percentage of age-matched controls, and data are presented as mean ± SEM of 7 animals. (C) The distribution of dynein in hippocampal subregions of control and STZ-induced diabetic animals was also analyzed by immunohistochemistry. The preparations were visualized in a laser scanning confocal microscope LSM 710 META (Zeiss, Germany). Scale bar: 50 µm. Insets shows the expression pattern of dynein in hippocampal slices. The quantification of immunoreactivity (fluorescence intensity arbitrary units) was performed and presented below the images.

Dynein is the major molecular motor protein that moves cargoes such as mitochondria, organelles and proteins towards the minus end of microtubules, thus being responsible for retrograde transport in neurons. In hippocampus, dynein mRNA and protein levels remained similar to those found in control samples at 2 and 8 weeks of diabetes (Figure 3A and B). Similarly, no changes were detected in CA1, CA3 and DG hippocampal subregions by immunohistochemistry in both time-points of diabetes, 2 and 8 weeks (Figure 3C).

### High Glucose Induces a Mild Decrease in KIF5B, but does not Affect the Overall Content and Distribution of Tau, KIF1A and Dynein in Hippocampal Cultures

**Figure 4:**
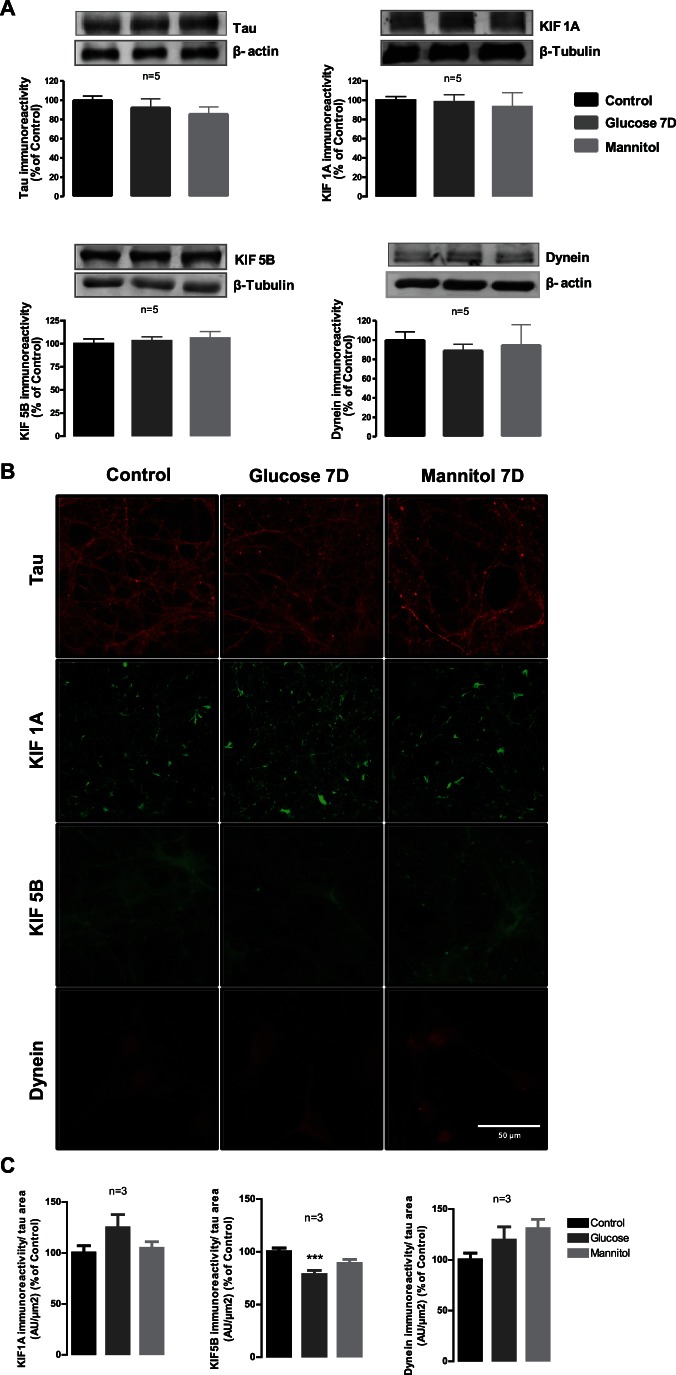
High glucose induces a mild decrease in KIF5B but does not affect the overall content and distribution of tau, KIF1A and dynein in hippocampal cultures. Cultured hippocampal neurons were exposed to 25 mM glucose (Control), 50 mM glucose (Glucose) and 25 mM mannitol (Mannitol) for 7 days. (A) The protein levels of tau, KIF1A, KIF5B and dynein were analyzed by western blotting. Representative images of protein immunoreactive bands are presented above the graphs, with the respective loading control (β-actin or β-III tubulin). The densitometry of each band was analyzed and the results are expressed as percentage of control ± SEM of 5 independent experiments. Statistical significance for the analysis of hippocampal cell cultures protein content was determined by using ANOVA, followed by Dunnett’s post hoc test. Differences were considered significant for *p*<0.05. (B) The immunoreactivity of KIF1A, KIF5B and dynein in the overall culture was analyzed by immunocytochemistry. Magnification 630×; Scale bar 50 µm. Quantification of the ratio between the fluorescence intensity for motor proteins (KIF1A, KIF5B or dynein) and the area of tau was performed for 3 independent experiments. *** p<0.001, significantly different from control as determined by one-way-ANOVA followed by Dunnet's post hoc test.

Hyperglycemia has been considered the main pathogenic factor underlying the development of diabetic complications, triggering several processes that may induce cell dysfunction. Here, we evaluated whether high glucose *per se*, mimicking hyperglycemic conditions, changes the content of proteins involved in axonal transport in hippocampal neuronal cultures. Exposure of hippocampal neurons to high glucose, or mannitol (osmotic control), did not affect the total protein content of tau, KIF1A, KIF5B and dynein, as it can be observed by western blotting in Figure 4A. By immunocytochemistry, we can observe that high glucose and mannitol did not induce any significant change in the distribution and content of KIF1A and dynein in the overall culture when compared to control conditions (Figure 4B). However, a significant decrease in the fluorescence intensity of KIF5B per tau area was found in hippocampal neurons exposed to elevated glucose (78.5±3.9% of control, Figure 4B). For both KIF1A and KIF5B, no changes in the content of anterograde motor proteins were observed in the osmotic control (mannitol).

### High Glucose Levels alter the Content and Distribution of Anterograde Motor Proteins in Axons

**Figure 5:**
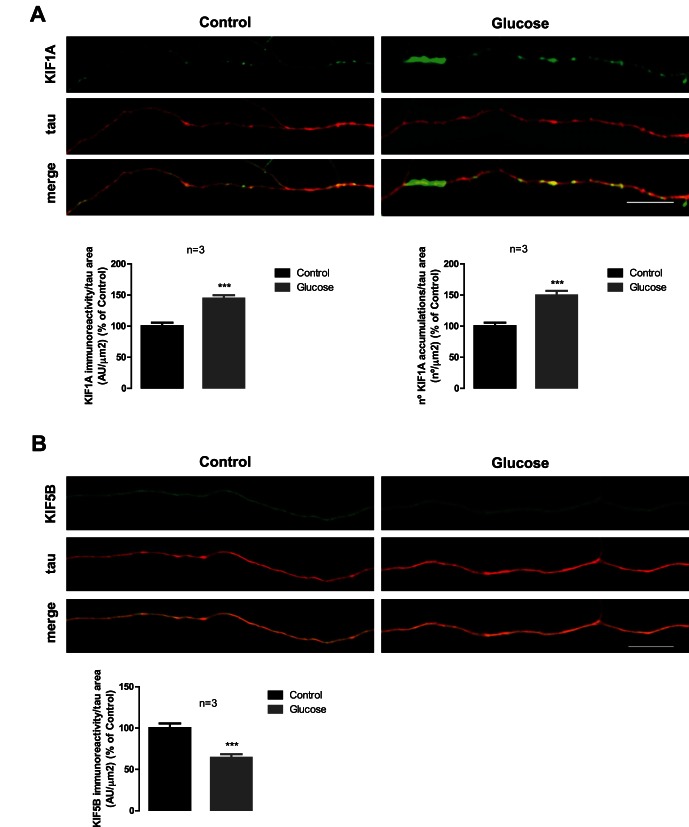
High glucose changes the number of KIF1A accumulated particles and KIF1A and KIF5B intensity in the axons of hippocampal neurons. Cultured hippocampal neurons were exposed to 25 mM glucose (Control) and 50 mM glucose (Glucose) for 7 days. The immunoreactivity and accumulated particles of KIF1A (A) and KIF5B (B), was analyzed by immunocytochemistry. Magnification 630×; Scale bar 10 µm. Quantification of accumulated particles and immunoreactivity was made for 3 independent cultures. *** p<0.001, significantly different from control as determined by the unpaired Student’s *t*-test.

Although only mild (KIF5B) or no changes (KIF1A) were detected in the content of motor proteins in hippocampal cultures exposed to high glucose levels, changes can be occurring specifically in axons and so being masked in the overall context. In fact, previous data obtained in our lab show that diabetic conditions affects the content of several presynaptic proteins in nerve terminals [2,5,6], thus suggesting that their transport to those distal sites might be impaired. Taken this into account, we hypothesized that exposure to high glucose induces changes in motor proteins specifically along axons. For that, we evaluated their content and distribution in the axons of hippocampal neurons. Exposure of hippocampal neurons to elevated glucose for 7 days increased the immunoreactivity of KIF1A in axons (KIF1A intensity/axonal area; increase to 144.3±5.5% compared to control conditions; Figure 5A). As it was already reported [20], and as it can be observed in the axon's segments shown in Figure 5A, KIF1A has a characteristic punctate pattern along axons of hippocampal neurons. To investigate whether high glucose levels would affect this distribution pattern, we measured the number of KIF1A accumulations along axons. An increase to 149.4±7.4% of control in the number of KIF1A accumulations per axonal area compared to control conditions was observed (Figure 5A). Also evident was a decrease (64.1±4.3% of control, Figure 5B) of KIF5B intensity in the axons of hippocampal cells incubated with high glucose for 7 days. These results are in agreement with the decrease observed in Figure 4B, thus showing that changes in the content of anterograde motor proteins are more evident when looking specifically to distant isolated axons. Due to the weak signal for dynein in axons, it was not possible to quantify this protein in hippocampal axons. These results show that hyperglycemia has a considerable effect on the axonal content and distribution of motor proteins involved in the anterograde transport.

### High Glucose Decreases SNAP-25 and Synaptophysin Immunoreactivity and Increases the Number of Accumulations of Synaptotagmin-1 in Hippocampal Axons

**Figure 6:**
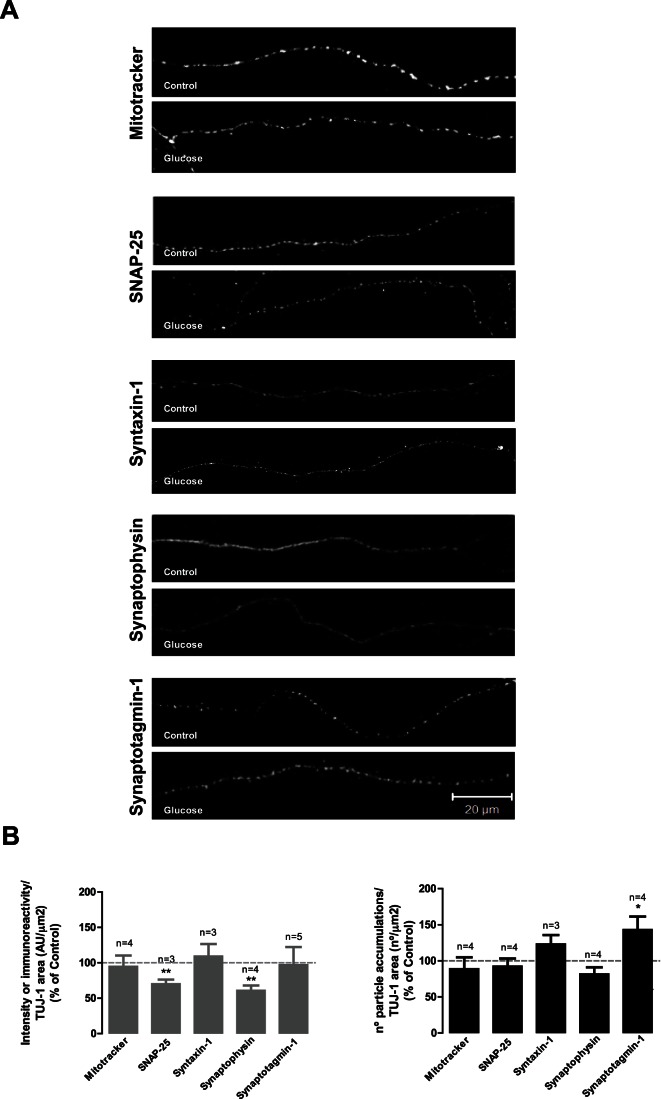
High glucose decreases SNAP-25 and synaptophysin immunoreactivity and increases the number of synaptotagmin-1 fluorescent accumulations. Cultured hippocampal neurons were exposed to 25 mM glucose (Control) and 50 mM glucose (Glucose) for 7 days. (A) The fluorescence intensity and the number of fluorescent accumulations related to mitochondria and synaptic proteins were analyzed in the axons of hippocampal neurons. The preparations were visualized under a laser scanning confocal microscope LSM 710 META (Zeiss, Germany). Scale bar: 20 µm. (B) The quantification of the number of fluorescent accumulations and intensity of fluorescence of mitochondria or immunoreactivity of synaptic proteins was performed and was expressed as percentage of the control..*p<0.05, **p<0.01, significantly different from control as determined by the unpaired Student’s *t*-test.

KIF1A is a neuron-specific kinesin motor protein that transports synaptic vesicle precursors containing synaptophysin and synaptotagmin, whereas KIF5B is known to transport mitochondria and synaptic vesicle precursors containing syntaxin-1 and SNAP-25 [19]. Since we detected changes in the number of KIF1A accumulations and changes in KIF1A and KIF5B immunoreactivity in hippocampal axons, we evaluated the effect of elevated glucose on the immunoreactivity/intensity of fluorescence and on the number of accumulations of synaptic proteins and mitochondria (stained with mitotracker, a fluorescent dye that stains mitochondria in live cells; Invitrogen, Life Technologies, Scotland, UK). No significant changes were observed in the intensity of fluorescence or in the number of fluorescent accumulations related to mitochondria in the axons of cultured hippocampal neurons exposed to high glucose compared to control (Figure 6A and B). Nevertheless, regarding synaptic proteins, we found that SNAP-25 and synaptophysin immunoreactivity was significantly decreased (70.5±5.8% and 61.1±6.8% of the control, respectively) in the axons of hippocampal neurons exposed to elevated glucose (Fig. 6A and B). Concerning the number of fluorescent accumulations of synaptic proteins in the axons of hippocampal neurons exposed to elevated glucose, we found that the number of accumulations of SNAP-25, syntaxin-1 and synaptophysin were similar to the control condition, with the exception of synaptotagmin-1, since the number of accumulations of this protein was significantly increased to 143.2±18.1% of the control.

## Discussion

In the present study, we addressed whether early diabetes can affect axonal motor proteins that are important for adequate transport of synaptic proteins and mitochondria in the hippocampus. Specifically, we showed that diabetes alters the mRNA levels, and immunoreactivity of KIF1A and KIF5B motor proteins in the hippocampus of diabetic rats. Moreover, in hippocampal neuronal cultures, we demonstrated that elevated glucose is able to change the immunoreactivity and number of fluorescent accumulations of motor and synaptic proteins in axons.

Due to their high polarity, neurons are particularly dependent on active intracellular transport. Deficits in this transport have been considered to contribute to the pathogenesis of multiple neurodegenerative diseases [21]. Direct evidence from genetic studies demonstrates that mutations in major components of the cytoskeleton and axonal transport result in axonal defects in Charcot-Marie-Tooth disease, amyotrophic lateral sclerosis and Alzheimer disease [7]. Post-translational modifications of cytoskeleton proteins also result in axonal defects in diabetic neuropathy [22]. In previous studies, we found that diabetes changes the levels of several synaptic proteins involved in exocytosis in hippocampal nerve terminals, with no changes in total extracts, suggesting that axonal transport of those proteins to distal synaptic sites may be impaired in diabetes [2,5]. In this study, we found that there is an increase in KIF1A and KIF5B levels in the hippocampus at 8 weeks after the onset of diabetes, with no changes in dynein levels, suggesting that the anterograde transport may be impaired in the hippocampus. An impairment of axonal transport of certain cargoes may lead to their accumulation in the cell body. In a rat model of *α*-synucleinopathy, elevated levels of KIF1A were observed in substantia nigra [23], and the authors suggested the possibility that accumulation of these motor proteins may be due to the imbalance in protein degradation and synthesis or to axonal transport deficit. Moreover, we found that long-term exposure to elevated glucose induces an accumulation of syntaxin-1, synaptotagmin-1 and VGluT-1 in the cell bodies of cultured hippocampal neurons [6], further suggesting that axonal transport may be impaired. These observations directed us to further analyze the effect of hyperglycemia, which is considered the main factor underlying the development of diabetic complications, on motor proteins, namely KIF1A, KIF5B and dynein. KIF1A transports synaptic vesicle precursors of synaptophysin and synaptotagmin-1, but does not transport organelles that contain plasma membrane proteins, such as syntaxin-1 or SNAP-25. These are transported by KIF5 motors. The number of fluorescent accumulations of KIF1A increased in the axons of hippocampal neurons exposed to elevated glucose for 7 days. Likewise, increased number of accumulations of synaptotagmin-1 was also detected. The accumulation of these particles may be due to impairments at the microtubule network and/or impairment in KIF1A motor function, leading to the accumulation of KIF1A.

KIF5B protein immunoreactivity in the axons of hippocampal cells incubated with high glucose for 7 days decreased and similarly SNAP-25 immunoreactivity was also decreased. Likewise, in our previous studies, a significant decrease in the content of SNAP-25 was detected in hippocampal cultures [6], as we had also demonstrated in hippocampal nerve terminals from diabetic rats [2]. These observations suggest that SNAP-25 appears to be particularly affected by hyperglycemic conditions, at least in hippocampal neurons, but the mechanisms underlying these effects are unknown. The reduction in SNAP-25 levels might significantly impair neurotransmission. In SNAP-25 KO neuronal cultures neurotransmitter release is almost abolished [24]. Moreover, synaptophysin immunoreactivity decreased in axons of hippocampal neurons exposed to high glucose. When analyzing the whole distribution of synaptophysin in hippocampal cultures [6], we did not detect any change in the immunoreactivity of this protein, but when we analyzed potential changes at hippocampal axons, a significant decrease in the immunoreactivity of this protein was detected. Since synaptophysin is an integral protein of the synaptic vesicle membranes that has been correlated with synaptic density and neurotransmitter release, this decrease may contribute to impair neurotransmitter release. The changes reported here in motor proteins, specifically those occurring in axons, namely the increase in the number of fluorescent accumulations of KIF1A and decreased immunoreactivity of KIF5B strongly suggest that axonal transport may be compromised. As a consequence, a decrease in the number of synaptic vesicles and synaptic density may ultimately account for changes in synaptic transmission in the hippocampus. Nevertheless, we must also keep in mind that other KIFs might partially compensate for the function of the kinesins here studied, and synaptic vesicle precursors might be transported by KIFs other than KIF1A and KIF5B [25]. Moreover, previous studies have shown that under diabetes, axonal transport defects may be due to altered levels of cargoes, as a result of decreased synaptic protein synthesis, abnormal translational processing of the protein or impaired incorporation of synaptic proteins into vesicles showing that impaired axonal transport of proteins is observed, while the actual rates of the axonal transport process are not affected [26–29].

Retrograde transport is powered mainly by cytoplasmic dynein, but some kinesins can also be involved in this transport [19]. KIF1A is responsible for the transport of dense-core vesicles in the axons of hippocampal neurons, remaining associated with dense-core vesicles during retrograde axonal transport, demonstrating that these vesicles retain the molecular machinery necessary for transport in both directions [30]. Defects in axonal transport of synaptophysin-containing vesicle precursors have been observed in KIF1A mutant mice [25]. Recent findings also demonstrate that KIF1A is necessary for BDNF-mediated hippocampal synaptogenesis and learning enhancement induced by environmental enrichment [31], which reinforces the importance of this kinesin in the hippocampus.

Tau is a microtubule associated protein, whose main function is to modulate the stability of axonal microtubules. Excessive tau phosphorylation is known to disrupt its binding to microtubules altering molecular trafficking, which ultimately may lead to synaptic dysfunction [32,33]. Diabetes induces abnormal hyperphosphorylation of tau in the brain, including the hippocampus [34], and proteolytic tau cleavage [35], both of which are associated with Alzheimer's disease [36]. In fact, tau modification can be induced by insulin dysfunction and hyperglycemia, which may contribute to the increased incidence of Alzheimer's disease in diabetic patients [37]. We did not detect any change in tau immmunoreactivity in hippocampal cultures exposed to high glucose. However, we cannot exclude the possibility of changes in tau phosphorylation state. Evidence obtained in kinesin-1 deficient mice suggested that defects in axonal transport can initiate biochemical changes that induce the activation of axonal stress kinase pathways leading to abnormal tau hyperphosphorylation. This further impairs axonal transport by disrupting the microtubule network and blocking axonal highways that ultimately will give rise to compromised synapse function and neurodegeneration [38,39].

KIF5 motors are also responsible for axonal transport of mitochondria. In KIF5A^−/−^ neurons, the velocity of mitochondrial transport is reduced both in anterograde and retrograde direction [40]. Decreased number of mitochondria in axons will likely decrease ATP supply to molecular motors leading to decreased anterograde and retrograde movement of both mitochondria and vesicles [7]. Growing evidence suggests that mitochondrial dysfunction play a significant role in neurodegenerative diseases like Huntington's disease, Alzheimer's disease and amyotrophic lateral sclerosis [41–43]. Mitochondrial dysfunction has also been proposed as a mediator of neurodegeneration in diabetes [44] but as far as we are concerned there are no studies addressing the effect of diabetes in mitochondria transport in the central nervous system. In our work, we did not detect changes in the intensity of fluorescence, neither in distribution or number of accumulations related with mitochondria in the axons of hippocampal neurons exposed to elevated glucose when compared to control. Nevertheless, we cannot exclude the possibility that hippocampal axons are being affected by diabetes since probably other factors, besides hyperglycemia, may also have an effect in mitochondria transport.

Hyperglycemia appears to be an important determinant for the changes observed in this study. However, under diabetic conditions, the lack or reduced levels of insulin, a potent trophic factor, might also play an important role in axonal transport impairment and synaptic changes observed in diabetic animals [45,46], thus contributing to changes in hippocampal physiology. For instance, short-term replacement of insulin in type I diabetic rats has shown to prevent cognitive deficits [47].

Inflammation may also be a factor contributing to changes in axonal transport in diabetes. Previously, it was reported that pro-inflammatory cytokines, such as tumor necrosis factor-α (TNF-α) and interleukin-1β, are upregulated in the hippocampus of diabetic BB/Wor rats [48] and STZ-induced diabetic animals [49]. TNF induces perinuclear clustering of mitochondria caused by impaired kinesin-mediated transport [50] and the activation of TNF receptor-1 induces the activation of kinase pathways, resulting in hyperphosphorylation of kinesin light chain (KLC) and inhibition of kinesin activity, evidencing direct regulation of kinesin-mediated organelle transport by extracellular stimuli via cytokine receptor signaling pathways in L929 cells [51]. Moreover, it was previously demonstrated that nitric oxide released from activated microglia inhibits axonal movement of synaptic vesicle precursors containing synaptophysin and synaptotagmin in hippocampal neurons, suggesting that disturbance of axonal transport by microglial nitric oxide may therefore be responsible for axonal injury and synaptic dysfunction in brain diseases characterized by neuroinflammation [52]. TNF produced by activated glial cells in inflammatory or degenerative neurological diseases affects neurites by acting on the kinesin-tubulin complex and inhibiting axonal mitochondria and synaptophysin transport via JNK in hippocampal cultures [53]. Very recently, it was demonstrated that hydrogen peroxide, a common reactive oxygen species elevated during inflammation, also inhibits axonal transport in hippocampal cultures [54]. Further studies will be needed to determine if similar pathways may be active under diabetic conditions, therefore contributing for the detected changes in the levels of synaptic and motor proteins in the hippocampus.

In summary, our data demonstrate that the mRNA levels and content of KIF1A and KIF5B motor proteins are altered in the hippocampus of diabetic rats. Furthermore, we showed that high glucose leads to an increase in the number of fluorescent accumulations of KIF1A and synaptotagmin-1 and decreased immunoreactivity of KIF5B, SNAP-25 and synaptophysin specifically in the axons of hippocampal neurons. Altogether, these changes suggest that the anterograde axonal transport may be impaired in the hippocampus, which may lead to changes in the content of synaptic proteins in nerve terminals, since their transport is mediated by these kinesins, and ultimately contribute to neural changes underlying diabetic encephalopathy.

## References

[1] Grillo CA, Piroli GG, Wood GE, Reznikov LR, McEwen BS, et al (2005) Immunocytochemical analysis of synaptic proteins provides new insights into diabetes-mediated plasticity in the rat hippocampus. Neuroscience 136: 477–486. 16226381 10.1016/j.neuroscience.2005.08.019

[2] Gaspar JM, Baptista FI, Galvao J, Castilho AF, Cunha RA, et al (2010a) Diabetes differentially affects the content of exocytotic proteins in hippocampal and retinal nerve terminals. Neuroscience 169: 1589–1600. 20600668 10.1016/j.neuroscience.2010.06.021

[3] Biessels GJ, Kamal A, Ramakers GM, Urban IJ, Spruijt BM, et al (1996) Place learning and hippocampal synaptic plasticity in streptozotocin-induced diabetic rats. Diabetes 45: 1259–1266. 8772732 10.2337/diab.45.9.1259

[4] Kamal A, Biessels GJ, Urban IJ, Gispen WH (1999) Hippocampal synaptic plasticity in streptozotocin-diabetic rats: impairment of long-term potentiation and facilitation of long-term depression. Neuroscience 90: 737–745. 10218775 10.1016/s0306-4522(98)00485-0

[5] Baptista FI, Gaspar JM, Cristovao A, Santos PF, Kofalvi A, et al (2011) Diabetes induces early transient changes in the content of vesicular transporters and no major effects in neurotransmitter release in hippocampus and retina. Brain Res 1383: 257–269. 21281613 10.1016/j.brainres.2011.01.071

[6] Gaspar JM, Castilho A, Baptista FI, Liberal J, Ambrosio AF (2010b) Long-term exposure to high glucose induces changes in the content and distribution of some exocytotic proteins in cultured hippocampal neurons. Neuroscience 171: 981–992. 20950673 10.1016/j.neuroscience.2010.10.019

[7] De Vos KJ, Grierson AJ, Ackerley S, Miller CC (2008) Role of axonal transport in neurodegenerative diseases. Annu Rev Neurosci 31: 151–173. 18558852 10.1146/annurev.neuro.31.061307.090711

[8] Lee PG, Hohman TC, Cai F, Regalia J, Helke CJ (2001) Streptozotocin-induced diabetes causes metabolic changes and alterations in neurotrophin content and retrograde transport in the cervical vagus nerve. Exp Neurol 170: 149–161. 11421592 10.1006/exnr.2001.7673

[9] Lee PG, Cai F, Helke CJ (2002) Streptozotocin-induced diabetes reduces retrograde axonal transport in the afferent and efferent vagus nerve. Brain Res 941: 127–136. 12031555 10.1016/s0006-8993(02)02645-8

[10] Jakobsen J, Brimijoin S, Skau K, Sidenius P, Wells D (1981) Retrograde axonal transport of transmitter enzymes, fucose-labeled protein, and nerve growth factor in streptozotocin-diabetic rats. Diabetes 30: 797–803. 7274587 10.2337/diab.30.10.797

[11] Medori R, Autilio-Gambetti L, Monaco S, Gambetti P (1985) Experimental diabetic neuropathy: impairment of slow transport with changes in axon cross-sectional area. Proc Natl Acad Sci U S A 82: 7716–7720. 2415969 10.1073/pnas.82.22.7716 PMC391404

[12] Yagihashi S, Kamijo M, Watanabe K (1990) Reduced myelinated fiber size correlates with loss of axonal neurofilaments in peripheral nerve of chronically streptozotocin diabetic rats. Am J Pathol 136: 1365–1373. 2141449 PMC1877565

[13] Zhang L, Ino-ue M, Dong K, Yamamoto M (2000) Retrograde axonal transport impairment of large- and medium-sized retinal ganglion cells in diabetic rat. Curr Eye Res 20: 131–136. 10617915

[14] Ino-Ue M, Zhang L, Naka H, Kuriyama H, Yamamoto M (2000) Polyol metabolism of retrograde axonal transport in diabetic rat large optic nerve fiber. Invest Ophthalmol Vis Sci 41: 4055–4058. 11095594

[15] Fernandez DC, Pasquini LA, Dorfman D, Aldana Marcos HJ, Rosenstein RE (2012) Early distal axonopathy of the visual pathway in experimental diabetes. Am J Pathol 180: 303–313. 22079928 10.1016/j.ajpath.2011.09.018 PMC3244601

[16] Sharma R, Buras E, Terashima T, Serrano F, Massaad CA, et al (2010) Hyperglycemia induces oxidative stress and impairs axonal transport rates in mice. PLoS One 5: e13463. 20976160 10.1371/journal.pone.0013463 PMC2956689

[17] Andersen CL, Jensen JL, Orntoft TF (2004) Normalization of real-time quantitative reverse transcription-PCR data: a model-based variance estimation approach to identify genes suited for normalization, applied to bladder and colon cancer data sets. Cancer Res 64: 5245–5250. 15289330 10.1158/0008-5472.CAN-04-0496

[18] Livak KJ, Schmittgen TD (2001) Analysis of relative gene expression data using real-time quantitative PCR and the 2(-Delta Delta C(T)) Method. Methods 25: 402–408. 11846609 10.1006/meth.2001.1262

[19] Hirokawa N, Noda Y, Tanaka Y, Niwa S (2009) Kinesin superfamily motor proteins and intracellular transport. Nat Rev Mol Cell Biol 10: 682–696. 19773780 10.1038/nrm2774

[20] Lee JR, Shin H, Ko J, Choi J, Lee H, et al (2003) Characterization of the movement of the kinesin motor KIF1A in living cultured neurons. J Biol Chem 278: 2624–2629. 12435738 10.1074/jbc.M211152200

[21] Chevalier-Larsen E, Holzbaur EL (2006) Axonal transport and neurodegenerative disease. Biochim Biophys Acta 1762: 1094–1108. 16730956 10.1016/j.bbadis.2006.04.002

[22] McLean WG (1997) The role of axonal cytoskeleton in diabetic neuropathy. Neurochem Res 22: 951–956. 9239750 10.1023/a:1022466624223

[23] Chung CY, Koprich JB, Siddiqi H, Isacson O (2009) Dynamic changes in presynaptic and axonal transport proteins combined with striatal neuroinflammation precede dopaminergic neuronal loss in a rat model of AAV alpha-synucleinopathy. J Neurosci 29: 3365–3373. 19295143 10.1523/JNEUROSCI.5427-08.2009 PMC2693917

[24] Bronk P, Deak F, Wilson MC, Liu X, Sudhof TC, et al (2007) Differential effects of SNAP-25 deletion on Ca2+ -dependent and Ca2+ -independent neurotransmission. J Neurophysiol 98: 794–806. 17553942 10.1152/jn.00226.2007

[25] Yonekawa Y, Harada A, Okada Y, Funakoshi T, Kanai Y, et al (1998) Defect in synaptic vesicle precursor transport and neuronal cell death in KIF1A motor protein-deficient mice. J Cell Biol 141: 431–441. 9548721 10.1083/jcb.141.2.431 PMC2148442

[26] Calcutt NA, Tomlinson DR, Willars GB, Keen P (1990) Axonal transport of substance P-like immunoreactivity in ganglioside-treated diabetic rats. J Neurol Sci 96: 283–291. 1695917 10.1016/0022-510x(90)90139-e

[27] Abbate SL, Atkinson MB, Breuer AC (1991) Amount and speed of fast axonal transport in diabetes. Diabetes 40: 111–117. 1707837 10.2337/diab.40.1.111

[28] Delcroix JD, Michael GJ, Priestley JV, Tomlinson DR, Fernyhough P (1998) Effect of nerve growth factor treatment on p75NTR gene expression in lumbar dorsal root ganglia of streptozocin-induced diabetic rats. Diabetes 47: 1779–1785. 9792548 10.2337/diabetes.47.11.1779

[29] Hellweg R, Raivich G, Hartung HD, Hock C, Kreutzberg GW (1994) Axonal transport of endogenous nerve growth factor (NGF) and NGF receptor in experimental diabetic neuropathy. Exp Neurol 130: 24–30. 7821393 10.1006/exnr.1994.1181

[30] Lo KY, Kuzmin A, Unger SM, Petersen JD, Silverman MA (2011) KIF1A is the primary anterograde motor protein required for the axonal transport of dense-core vesicles in cultured hippocampal neurons. Neurosci Lett 491: 168–173. 21256924 10.1016/j.neulet.2011.01.018

[31] Kondo M, Takei Y, Hirokawa N (2012) Motor protein KIF1A is essential for hippocampal synaptogenesis and learning enhancement in an enriched environment. Neuron 73: 743–757. 22365548 10.1016/j.neuron.2011.12.020

[32] Ebneth A, Godemann R, Stamer K, Illenberger S, Trinczek B, et al (1998) Overexpression of tau protein inhibits kinesin-dependent trafficking of vesicles, mitochondria, and endoplasmic reticulum: implications for Alzheimer's disease. J Cell Biol 143: 777–794. 9813097 10.1083/jcb.143.3.777 PMC2148132

[33] Obulesu M, Venu R, Somashekhar R (2011) Tau mediated neurodegeneration: an insight into Alzheimer's disease pathology. Neurochem Res 36: 1329–1335. 21509508 10.1007/s11064-011-0475-5

[34] Qu Z, Jiao Z, Sun X, Zhao Y, Ren J, et al (2011) Effects of streptozotocin-induced diabetes on tau phosphorylation in the rat brain. Brain Res 1383: 300–306. 21281610 10.1016/j.brainres.2011.01.084

[35] Kim B, Backus C, Oh S, Hayes JM, Feldman EL (2009) Increased tau phosphorylation and cleavage in mouse models of type 1 and type 2 diabetes. Endocrinology 150: 5294–5301. 19819959 10.1210/en.2009-0695 PMC2795717

[36] Iqbal K, Liu F, Gong CX, Alonso Adel C, Grundke-Iqbal I (2009) Mechanisms of tau-induced neurodegeneration. Acta Neuropathol 118: 53–69. 19184068 10.1007/s00401-009-0486-3 PMC2872491

[37] Liu Y, Liu F, Grundke-Iqbal I, Iqbal K, Gong CX (2011) Deficient brain insulin signalling pathway in Alzheimer's disease and diabetes. J Pathol 225: 54–62. 21598254 10.1002/path.2912 PMC4484598

[38] Falzone TL, Stokin GB, Lillo C, Rodrigues EM, Westerman EL, et al (2009) Axonal stress kinase activation and tau misbehavior induced by kinesin-1 transport defects. J Neurosci 29: 5758–5767. 19420244 10.1523/JNEUROSCI.0780-09.2009 PMC3849468

[39] Falzone TL, Gunawardena S, McCleary D, Reis GF, Goldstein LS (2010) Kinesin-1 transport reductions enhance human tau hyperphosphorylation, aggregation and neurodegeneration in animal models of tauopathies. Hum Mol Genet 19: 4399–4408. 20817925 10.1093/hmg/ddq363 PMC2957317

[40] Karle KN, Mockel D, Reid E, Schols L (2012) Axonal transport deficit in a KIF5A(−/− ) mouse model. Neurogenetics 13: 169–179. 22466687 10.1007/s10048-012-0324-y PMC3332386

[41] Brownlees J, Ackerley S, Grierson AJ, Jacobsen NJ, Shea K, et al (2002) Charcot-Marie-Tooth disease neurofilament mutations disrupt neurofilament assembly and axonal transport. Hum Mol Genet 11: 2837–2844. 12393795 10.1093/hmg/11.23.2837

[42] Stamer K, Vogel R, Thies E, Mandelkow E, Mandelkow EM (2002) Tau blocks traffic of organelles, neurofilaments, and APP vesicles in neurons and enhances oxidative stress. J Cell Biol 156: 1051–1063. 11901170 10.1083/jcb.200108057 PMC2173473

[43] Reddy PH (2011) Abnormal tau, mitochondrial dysfunction, impaired axonal transport of mitochondria, and synaptic deprivation in Alzheimer's disease. Brain Res 1415: 136–148. 21872849 10.1016/j.brainres.2011.07.052 PMC3176990

[44] Fernyhough P, Roy Chowdhury SK, Schmidt RE (2010) Mitochondrial stress and the pathogenesis of diabetic neuropathy. Expert Rev Endocrinol Metab 5: 39–49. 20729997 10.1586/eem.09.55 PMC2924887

[45] Sima AA, Li ZG (2005) The effect of C-peptide on cognitive dysfunction and hippocampal apoptosis in type 1 diabetic rats. Diabetes 54: 1497–1505. 15855338 10.2337/diabetes.54.5.1497

[46] Li ZG, Zhang W, Sima AA (2005) The role of impaired insulin/IGF action in primary diabetic encephalopathy. Brain Res 1037: 12–24. 15777748 10.1016/j.brainres.2004.11.063

[47] Biessels GJ, Kamal A, Urban IJ, Spruijt BM, Erkelens DW, et al (1998) Water maze learning and hippocampal synaptic plasticity in streptozotocin-diabetic rats: effects of insulin treatment. Brain Res 800: 125–135. 9685609 10.1016/s0006-8993(98)00510-1

[48] Sima AA, Zhang W, Kreipke CW, Rafols JA, Hoffman WH (2009) Inflammation in Diabetic Encephalopathy is Prevented by C-Peptide. Rev Diabet Stud 6: 37–42. 19557294 10.1900/RDS.2009.6.37 PMC2712918

[49] Kuhad A, Bishnoi M, Tiwari V, Chopra K (2009) Suppression of NF-kappabeta signaling pathway by tocotrienol can prevent diabetes associated cognitive deficits. Pharmacol Biochem Behav 92: 251–259. 19138703 10.1016/j.pbb.2008.12.012

[50] De Vos K, Goossens V, Boone E, Vercammen D, Vancompernolle K, et al (1998) The 55-kDa tumor necrosis factor receptor induces clustering of mitochondria through its membrane-proximal region. J Biol Chem 273: 9673–9680. 9545301 10.1074/jbc.273.16.9673

[51] De Vos K, Severin F, Van Herreweghe F, Vancompernolle K, Goossens V, et al (2000) Tumor necrosis factor induces hyperphosphorylation of kinesin light chain and inhibits kinesin-mediated transport of mitochondria. J Cell Biol 149: 1207–1214. 10851018 10.1083/jcb.149.6.1207 PMC2175118

[52] Stagi M, Dittrich PS, Frank N, Iliev AI, Schwille P, et al (2005) Breakdown of axonal synaptic vesicle precursor transport by microglial nitric oxide. J Neurosci 25: 352–362. 15647478 10.1523/JNEUROSCI.3887-04.2005 PMC6725475

[53] Stagi M, Gorlovoy P, Larionov S, Takahashi K, Neumann H (2006) Unloading kinesin transported cargoes from the tubulin track via the inflammatory c-Jun N-terminal kinase pathway. FASEB J 20: 2573–2575. 17068110 10.1096/fj.06-6679fje

[54] Fang C, Bourdette D, Banker G (2012) Oxidative stress inhibits axonal transport: implications for neurodegenerative diseases. Mol Neurodegener 7: 29. 22709375 10.1186/1750-1326-7-29 PMC3407799

